#  Raising Awareness of Sleep as a Healthy Behavior

**DOI:** 10.5888/pcd10.130081

**Published:** 2013-08-08

**Authors:** Geraldine S. Perry, Susheel P. Patil, Letitia R. Presley-Cantrell

**Affiliations:** Author Affiliations: Susheel P. Patil, Johns Hopkins Sleep Disorders Center, Baltimore, Maryland; Letitia R. Presley-Cantrell, Centers for Disease Control and Prevention, Atlanta, Georgia.

Sleep is an essential component of health, and its timing, duration, and quality are critical determinants of health (1). Sleep may play an important role in metabolic regulation, emotion regulation, performance, memory consolidation, brain recuperation processes, and learning (2). Because of the importance of these functions, sleep should be viewed as being as critical to health as diet and physical activity. However, public health practitioners and other health care providers have not focused major attention on the importance of sleep to health. In this essay, we briefly summarize the scientific literature about hours of sleep needed and why sleep is an important public health issue. We also suggest areas for expanding sleep research and strategies for increasing awareness of the importance of sleep and improving sleep health. Finally, we call for action to bring sleep to the forefront of public health.

## How Much Sleep is Needed and Are We There?

The 2006 Institute of Medicine (IOM) report *Sleep Disorders and Sleep Deprivation* indicates that the average basal sleep needs of adults is approximately 7 to 8 hours per night, and the optimal sleep duration for adolescents is 9 hours per night (1). However, more than 35% of adults report getting fewer than 7 hours of sleep during a 24-hour period (3,4), and almost 70% of high school students report getting fewer than 8 hours of sleep on an average weeknight (5). Overall, about 15 million children in the United States do not get sufficient sleep (6).

Among adults, the reasons for sleep loss appear to be related mainly to lifestyle, work schedules (shift work and long hours), or sleep disorders (1). Approximately 20% of workers are engaged in shift work, which often leads to longer work hours (1). Among adolescents, insufficient sleep is associated with greater use of social media technology, and among younger children it is associated with depressive symptomatology, family disagreements, and safety issues around home, school and neighborhood (6).

## Why Is Sleep a Public Health Issue?

Insufficient sleep has major health consequences in adults, adolescents, and young children. Strong evidence exists that among adults insufficient sleep has a significant effect on numerous health conditions, including chronic disease development and incidence (1). For instance, short sleep duration (<7 hours of sleep per night) and poor sleep quality are associated with cardiovascular morbidity and metabolic disorders such as glucose intolerance, which may lead to obesity, diabetes, heart disease, and hypertension (1). People who have short sleep duration are at 1.48 times greater risk of developing and dying of coronary heart disease than controls and 1.15 times more likely to have a stroke (7). Children who experience short sleep duration are more likely to become obese than those who do not (8).

Insufficient sleep also affects immunologic function and development of mood disorders and is associated with depression; deficits in cognition, memory and learning; and reduced quality of life (1). Adults who sleep fewer than 7 hours per night have greater difficulty concentrating, remembering, and performing other daily activities than those who sleep 7 to 9 hours a night (4). Children and adolescents who get insufficient sleep have impaired behavior, mood, and performance (9).

One major consequence of insufficient sleep is daytime sleepiness, which reduces alertness and causes slow reaction time, leading to occupational and medical errors, workplace injuries, impaired driving, and motor vehicle accidents (1). In 2009, almost 5% of adults in 12 states reported that during the previous 30 days they had nodded off or fallen asleep while driving (3). In 2005, drowsy driving contributed to 100,000 motor vehicle accidents and 15,000 deaths (10).

The public health burden of sleep deprivation is enormous. There are substantial public health investments in all areas related to sleep, from obesity and other chronic conditions to motor vehicle accidents. Insufficient sleep, unlike other health risk factors such as smoking, excessive alcohol consumption, obesity, and physical inactivity, has historically received much less attention in the public health and clinical settings. Insufficient sleep is an important public health risk factor that would benefit from further investigation.

## Lack of Awareness

Despite strong evidence of the relationship between insufficient sleep and health problems, most people are unaware of the amount of sleep they need, their level of sleep deprivation, and the negative impact of sleep deprivation on health. Because of lack of awareness, sleep is not commonly incorporated into public health approaches. In addition, many health care providers do not counsel their patients about healthy sleep habits (11). In a study of health care screening among 121 primary care clinics, only 43% included sleep-related questions on their screening batteries compared with 100% for smoking and alcohol, 93% for healthy eating, and 86% for physical activity (11). It is not clear why sleep is not included in health screenings, but it may be related to the clinician’s lack of knowledge of the importance of sleep. In 2002, only 10% of primary care providers described their knowledge of sleep and sleep disorders as good (12).

Although little evidence exists on the effectiveness of sleep screening and counseling on sleep behavior, screening and counseling has been shown to improve the health behaviors of patients in other areas, such as dietary habits, smoking cessation, and physical activity (13). Therefore, giving providers information about screening and counseling for appropriate sleep time and needs could better equip primary care and public health professionals with the knowledge needed to screen and counsel patients to promote sleep as a healthy behavior (1). However, further investigation is needed on the effectiveness of sleep screening and sleep counseling.

## Strategies to Improve Awareness and Sleep Health

Information about the physiology of sleep and sleep disorders is widely available, but much less work has been done on effective strategies to promote sleep as a healthy behavior. This field is prime for public health investigations and interventions to reduce the negative effect of insufficient sleep as a common risk factor for many health outcomes. Some suggested strategies for improving sleep initiation and sleep maintenance, duration, and quality are consistency in bedtime and rising; maintaining an appropriate sleeping environment (dark, relaxing, not too hot or cold); avoiding television -watching before bed, avoiding use of electronics or reading in the bedroom; and avoiding large meals and physical activity before going to bed (www.sleepfoundation.org). However, more research is needed to evaluate the effectiveness of these suggested strategies to improve sleep behavior and health.

The IOM report calls for several approaches to reduce the public health burden of insufficient sleep through increasing public awareness of the importance of sleep and improving diagnosis and treatment of sleep disorders (1). Reaching these goals will require 1) improved public education on the need for sleep and the consequences of insufficient sleep; 2) more training for public health professionals and health care providers on screening and counseling; and 3) improved evidence of the burden of insufficient sleep acquired through surveillance and monitoring tools.

Federal agencies, public health partners, and private organizations are collaborating to employ IOM strategies. For example, The National Sleep Awareness Roundtable (NSART) (www.nsart.org), a national coalition of government, professional, volunteer, and other organizations, is collaborating to raise awareness about sleep among the public, increase the understanding of the importance of sleep, and reduce the public health and safety impact of sleep deprivation and sleep disorders by improving communication and collaboration among local, state, and federal agencies. NSART member organizations have contributed to sleep awareness by providing training workshops on healthy sleep for primary care providers, by producing free local initiatives to educate primary care health providers on sleep and sleep disorders, by promoting Drowsy Driving Prevention Week and National Sleep Awareness Week to educate the public, and by publishing research findings. Several of the Centers for Disease Control and Prevention’s surveillance systems have added questions on sleep to provide state and national data on the burden of insufficient sleep (14–16); however, more national data are needed on young children (aged 0–12 years). The 2020 Health Objectives (17) added sleep as one of its new areas, focusing on increasing the proportion of adults and students in grades 9 through 12 who get sufficient sleep, decreasing the number of vehicular crashes resulting from drowsy driving, and increasing the proportion of persons with sleep apnea symptoms who are evaluated.

## Call to Action

Because of the lack of awareness of the benefits of healthy sleep, multisectoral public health campaigns, similar to those related to smoking cessation and reducing excessive alcohol consumption, are needed to educate the public about the importance of sleep and the consequences of insufficient sleep. Suggested strategies to improve sleep health include the following:

- Research on the effectiveness of screening and counseling efforts
- Education of employers on the health effects of long shifts and insufficient sleep
- Delaying school start time for high school students
- Educating the public on the risks of drowsy driving
- Improving surveillance of sleep health, especially among young children

Finally, the critical public health message is: Sleep is essential for good health; it is a necessity, not a luxury.
